# Crystal structure and Hirshfeld surface analysis of (*S*)-*N*-methyl-1-phenyl­ethan-1-aminium chloride

**DOI:** 10.1107/S2056989021013645

**Published:** 2022-01-07

**Authors:** Jan-Lukas Kirchhoff, Lukas Brieger, Carsten Strohmann

**Affiliations:** a Technische Universität Dortmund, Fakultät Chemie und Chemische Biologie, Otto-Hahn-Strasse 6, 44227 Dortmund, Germany

**Keywords:** crystal structure, chiral amines, Hirshfeld atom refinement (HAR), *NoSpherA2*, separation strategies, Hirshfeld surface analysis

## Abstract

The title compound C_9_H_14_N^+^·Cl^−^, (**1**), can be synthesized starting from (*S*)-*N*-methyl-1-phenyl­ethan-1-amine (**2**). Compound **2** upon addition of HCl·Et_2_O leads to crystallization of compound **1** as colorless blocks. The configuration of compound **1** is stable as well as preserved in space group *P*2_1_2_1_2_1_. Ammonium chlorides, like the title compound, are often observed as undesirable by-products in amino­silylation of chloro­silanes. Additionally, these by-products are usually soluble in selected organic solvents, which require difficult separation steps. Therefore, detailed studies on structural features and inter­molecular inter­actions performed by Hirshfeld atom refinement (HAR) using *NoSpherA2* [Kleemiss *et al.* (2021 ▸). *Chem. Sci.* **12**, 1675–1692] and Hirshfeld surface analysis were used to address structural issues on that separation problem.

## Abstract 2

Ammonium chlorides, such as the title compound (*S*)-*N*-methyl-1-phenyl­ethan-1-amnonium chloride (**1**), usually represent by-products in amination processes of chloro­silanes. Hirshfeld surface analysis and Hirshfeld atom refinement (HAR) were performed to obtain detailed information about the crystal packing and the exact position of the hydrogen atoms of compound **1**.

## Chemical context

Chiral amines represent a central role in synthetic chemistry, finding more and more applications in asymmetric syntheses (Liu *et al.*, 2020 ▸). In addition to asymmetric inductions on double bonds of organic mol­ecules, they also serve as amination reagents of chloro­silanes (Wannagat & Klemke, 1979 ▸; Veith, 1987 ▸). Next to meth­oxy­silanes, those chlorosilanes are the most important starting compounds for the synthesis of amino­silanes (Bauer & Strohmann, 2012 ▸). The title compound (*S*)-*N*-methyl-1-phenyl­ethan-1-aminium chloride (**1**), represents the ammonium chloride salt of (*S*)-*N*-methyl-1-phenyl­ethan-1-amine (**2**), which is often used as a chiral auxiliary in reagent inductions on prochiral silicon centers (Bauer & Strohmann, 2014 ▸). Compound **2** and its derivatives are characterized by well-known methods of enanti­omeric resolution (Ingersoll, 1937 ▸; Baltzly & Russell, 1953 ▸). The synthesis of Si–N-functionalized silanes starting from chloro­silanes in combination with amines is also very well known (Sakaba *et al.*, 2015 ▸; Zibula *et al.*, 2020 ▸). However, the formation of the undesirable ammonium chloride is often observed as a by-product, which is also soluble in small amounts of selected organic solvents. The corresponding reaction is shown in the scheme below. 
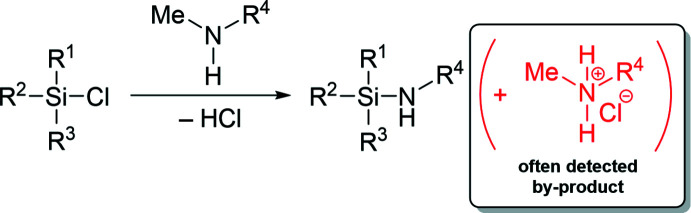


Compound **1** was crystallized for the first time and may be used to analyze supra­molecular inter­actions, in particular those which could be directly related to the aforementioned separation problem. To describe the positions of the hydrogen atoms as accurately as possible, all hydrogen atoms were refined anisotropically by *NoSpherA2* (Kleemiss *et al.*, 2021 ▸).

## Structural commentary

**Figure 1:**
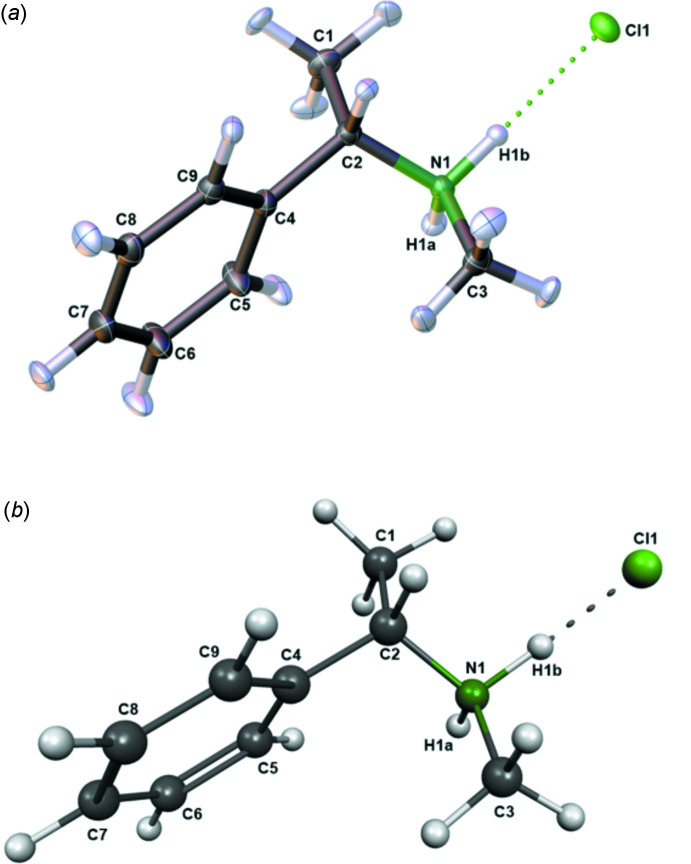
*(a)* The mol­ecular structure of **1** illustrated showing 50% displacement ellipsoids including. All hydrogen atoms except H1*b* were refined by Hirshfeld atom refinement (HAR) performed by *NoSpherA2* implementation in *OLEX2.* (*b*) Visualization of the calculated structure of compound **1** with *Molekel 4.3* performed at the M062X/6–31+G(*d*) levels.

Compound **1** crystallized from diethyl ether at room temperature in the shape of colorless blocks with ortho­rhom­bic (*P*2_1_2_1_2_1_) symmetry. The absolute configuration of the chiral ammonium chloride **1** in the measured crystal can be assigned with the (*S*)-configuration using the Cahn–Ingold–Prelog (CIP) prioritization (Cahn *et al.*, 1966 ▸); the Flack parameter amounts to −0.03 (3) (Flack, 1983 ▸). The mol­ecular structure of **1** is illustrated in Fig. 1 ▸. All hydrogen atoms except H1*b* were refined using *NoSpherA2* (Kleemiss *et al.*, 2021 ▸). No particularly large ellipsoids are observed here, whereas hydrogen atom H1*b* is highly deformed and distorted in anisotropic treatment. The substantial contribution from deformation of the electron density involving the chloride ion, which also includes polarization, may be responsible for the observed ellipsoidal shape of the hydrogen atom H1*b*. Thus it is difficult to deconvolute the effect of thermal motion in this inter­action and to model the same satisfactorily. Therefore, the hydrogen atom H1*b* was isotropically modeled for following analyses as shown in Fig. 1 ▸(*a*).

In the literature, known C*sp* ^3^—N bond lengths are in a range of 1.4816 (4) Å (N1—C3) and 1.5034 (4) Å (N1—C3), which are typical for most structurally analyzed ammonium salts (Allen *et al.*, 1987 ▸). To discuss the bond distances in the solid-state structure, quantum chemical calculations were performed at the level M062X/6-31+G(d), which gave comparable results. The mol­ecular structure of compound **1** in the gas phase is shown in Fig. 1 ▸(*b*). All conformations were taken from the solid-state structure at the start of the optimization. The result of the calculation provides smaller C*sp* ^3^—N bond lengths than from the solid-state structure in principle. The calculated bond lengths are 1.4762 Å (N1—C3) and 1.4946 Å (N1—C2).

Hydrogen-bond lengths as well as associated angles are shown in Table 1 ▸. The calculated hydrogen bond of 1.7051 Å (N1—H1*b*⋯Cl1) was not described sufficiently with the addition of the used potential and basis set. Therefore, a large deviation can be observed from the analyzed distance compared to the crystal structure. Further analyses concerning supra­molecular inter­actions are discussed in detail in the next section.

The stereogenic carbon center features a tetra­hedral geometry, which is slightly distorted as shown by the angle of 107.44 (2)° (C1—C2—N1). However, the geometric distortion of a tetra­hedral carbon center has been observed in many compounds with different substituents (Xu *et al.*, 2000 ▸).

## Supra­molecular features

**Figure 2:**
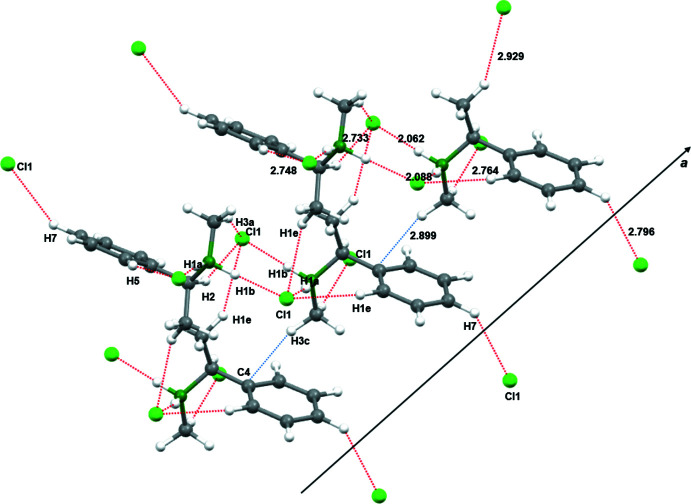
A view along the *a*-axis direction of the crystal packing of compound **1**. Selected hydrogen-bond lengths (in Å) are indicated.

**Figure 3:**
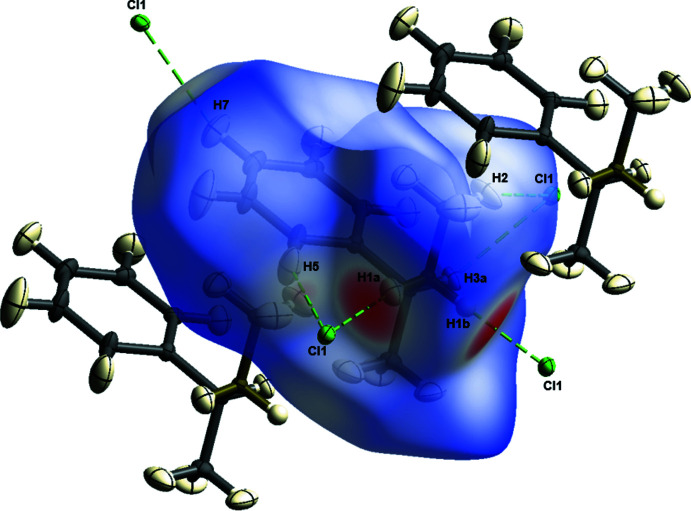
Hirshfeld surface of compound **1** generated by *CrystalExplorer21*.

**Figure 4:**
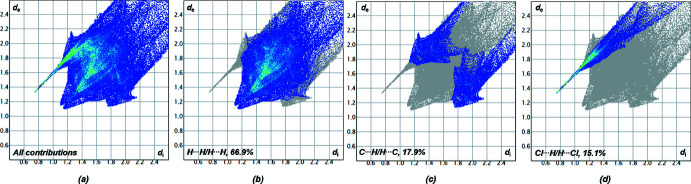
Two-dimensional fingerprint plots of compound **1** showing close contacts of (*a*) all contributions in the crystal and those delineated into (*b*) H⋯H, (*c*) C⋯H/H⋯C and (*d*) Cl⋯H/H⋯Cl-inter­actions. Symmetry codes: (i) 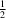 − *x*, −*y*, 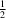 + *z*; (ii) −*x*, 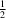 + *y*, 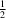 − *z*; (iii) 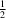 + *x*, 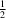 − *y*, −*z*.

The crystal packing along the *a*-axis of compound **1** is shown in Fig. 2 ▸. To analyze supra­molecular packing inter­actions in more detail, Hirshfeld surface analyses were performed. The Hirshfeld surface mapped over *d*_norm_ in the range from −0.5483 to 1.5337 arbitrary units generated by *CrystalExplorer2021* (Spackman *et al.*, 2021 ▸; Turner *et al.*, 2017 ▸) is shown in Fig. 3 ▸. Fingerprint plots, which are illustrated in Fig. 4 ▸, were also generated by *CrystalExplorer2021*. First, the crystal structure was analyzed to clarify for the influence of hydrogen bonds. Particularly noticeable on the Hirshfeld surface are C—H⋯Cl contacts, which are shown in red on the potential surface in Fig. 3 ▸.

The primary share of 66.9% can be assigned to weak van der Waals H⋯H contacts, which should play a minor role in terms of crystal packing. In contrast, Cl⋯H/H⋯Cl contacts in particular, which represent the smallest fraction of inter­actions (15.1%), however represent the most intense contacts on the surface (Fig. 4 ▸). Hydrogen bonds with a length up to 2.200 Å are shown in Table 1 ▸. The analysis of the hydrogen-bonding network leads to the result that all hydrogen bridges can be assigned to one graph-set motif. Both hydrogen bonds in Table 1 ▸ can be assigned *D*_1_^1^ (2) (Etter *et al.*, 1990 ▸).

In addition to the influence of C—H⋯Cl contacts, the influence of possible π-inter­actions was analyzed by *CrystalExplorer2021*. As can be seen in Fig. 2 ▸, compound **1** forms one-dimensional chains along the *a*-axis direction in the crystal structure. These can be attributed to the strong C—H⋯Cl inter­actions already mentioned, as well as additional C—H⋯π inter­actions, which are illustrated in Fig. 2 ▸. Consequently, these π-inter­actions could contribute a significant part to the crystal packing structure. However, C—H⋯π contacts are only weakly visible on the Hirshfeld surface.

## Database survey

There are some crystallographically characterized examples based on the structure of compound **1**. Depicted examples found in Cambridge Structural Database (WebCSD, November 2021; Groom *et al.*, 2016 ▸) include *N*,*N*′-bis­(1-phenyl­eth­yl)cyclo­hex-4-ene-1,2-diaminium dichloride monohydrate, C_22_H_32_N_2_O_2_Cl (CSD refcode KIZHIM; Savoia *et al.*, 2014 ▸), 2-(eth­oxy­carbon­yl)-*N*-(1-phenyl­eth­yl)cyclo­pent­an-1-aminium chloride, C_16_H_24_ClNO_2_ (BAJSIS; Lee *et al.*, 2021 ▸), *N*-[(*R*)-(cyclo­hexan-(*R*)-2-ol)]-(*R*)-a-methyl­benzyl ammonium chloride, C_14_H_22_NOCl (TAYWOF; Barbaro *et al.*, 1996 ▸), [2-(1*H*-inden-3-yl)eth­yl][(1*R*)-1-phenyl­eth­yl]ammo­nium chloride, C_19_H_22_NCl (GOGCUC; Ross *et al.*, 2015 ▸), *cis*-(a*R*,1*R*,2*S*)-2-meth­oxy-1-(1-phenyl­ethyl­amino)­cyclo­penta­ne­carboxamide hydro­chloride, C_15_H_23_N_2_O_2_Cl (NAFZIE; Meyer *et al.*, 2004 ▸). A comparison with the last two structures mentioned shows that compound **1** is characterized by partic­ularly short C—H⋯Cl (2.0-2.1 Å) hydrogen bonds. The smallest observed hydrogen bond of the two literature known compounds amounts to 2.2 Å. These longer distances could be due to the more sterically demanding substituents, which are less pronounced in compound **1**. Moreover, in terms of the crystal packing, the compounds do not exhibit one-dimensional chains, as C—H⋯π contacts were not observed. This unique feature of (*S*)-*N*-methyl-1-phenyl­ethan-1-aminium chloride (**1**), which does not appear in the literature compounds, could again be attributed to the steric crowding of the other compounds.

## Synthesis and crystallization

The reaction scheme for the synthesis of compound **1** is illustrated in the scheme below. (*S*)-*N*-methyl-1-phenyl­ethan-1-amine (**2**) (1.48 mmol) was dissolved in diethyl ether (5 mL). A 2 *M* solution of HCl in diethyl ether (1.78 mmol) was added dropwise. The solution was stored at 298 K for three days. Afterwards all volatile compounds were removed and the raw product was washed with cold *n*-pentane (1 ml). (*S*)-*N*-methyl-1-phenyl­ethan-1-aminium chloride (**1**) was isolated as colorless crystalline plates. 
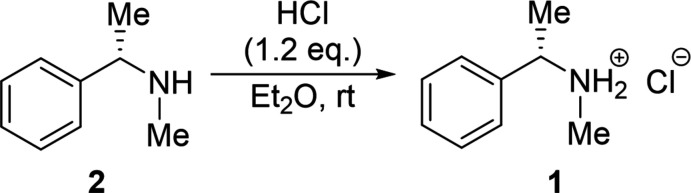


^1^H NMR (300.13 MHz, CDCl_3_): *δ* = 1.88 (*s*, 3H, CHC*H*_3_), 2.46 (*s*, 3H, NC*H*_3_), 1.65–1.90 (*br. s*, 1H, C*H*CH_3_), 7.38–7.45 (*m*, 3H, C*H*_ar, *meta*_, C*H*_ar, *para*_), 7.60 (*d*, 2H, ^3^ *J*_H–H_ = 3.9 Hz, C*H*_ar, *ortho*_), 9.78 (*br. s*, 1H, N*H*⋯Cl), 10.17 (*br. s*, 1H, N*H*⋯Cl) ppm.

{1H}^13^C NMR (100.64 MHz, CDCl_3_): *δ* = 20.5 (1C, CH*C*H_3_), 31.0 (1C, *C*HCH_3_), 60.2 (2C, N*C*H_3_), 127.9 (2C, *C_ortho_* H_ar_), 129.5 (2C, *C_meta_* H_ar_), 135.6 (1C, *C_para_* H_ar_) ppm.

CHN analysis: calculated: C = 62.97%, H = 8.22% N = 8.16%; Found: C = 62.6%, H = 7.9%, N = 7.8%.

*R*_f_: (CH_2_Cl_2_ / MeOH; 10:1) = 0.20.

## Refinement

Crystal data, data collection and structure refinement details are summarized in Table 2 ▸. All H atoms except H1*b* were refined freely using independent values of each *U*_iso_(H). Hirshfeld atom refinements (HAR; Fugel *et al.*, 2018 ▸) was performed with the *NoSpherA2* (Kleemiss *et al.*, 2021 ▸) implementation in *OLEX2* (Dolomanov *et al.*, 2009 ▸), using the restricted Khom–Sham method with the PBE-functional (Perdew *et al.*, 1996 ▸) and basis set def2-SVP (Weigend & Ahlrichs, 2005 ▸). For the HAR approach, all H atoms except H1*b* were refined anisotropically and independently. Atom H1*b* was refined freely without using HAR by *NoSpherA2*.

## Supplementary Material

Crystal structure: contains datablock(s) global, I. DOI: 10.1107/S2056989021013645/dx2041sup1.cif

Supporting information file. DOI: 10.1107/S2056989021013645/dx2041Isup2.cml

CCDC reference: 2132333

Additional supporting information:  crystallographic information; 3D view; checkCIF report

## Figures and Tables

**Table 1:** Hydrogen-bond geometry (Å, °)

*D*—H⋯*A*	*D*—H	H⋯*A*	*D*⋯*A*	*D*—H⋯*A*
N1—H1*a*⋯Cl1^i^	1.029 (6)	2.088 (6)	3.1075 (3)	170.4 (5)
N1—H1*b*⋯Cl1	1.031 (6)	2.062 (6)	3.0925 (4)	177.8 (5)

Symmetry code: (i) -x+1, y-{\script{1\over 2}}, -z+{\script{1\over 2}}.

**Table 2:** Experimental details

Crystal data	
Chemical formula	C_9_H_14_N^+^·Cl^−^
*M*_r_	171.67
Crystal system, space group	Orthorhombic, *P*2_1_2_1_2_1_
Temperature (K)	100
*a*, *b*, *c* (Å)	6.7723 (6), 7.1806 (5), 20.542 (2)
*V* (Å^3^)	998.96 (16)
*Z*	4
Radiation type	Mo *K*α
μ (mm^−1^)	0.32
Crystal size (mm)	0.48 × 0.39 × 0.37

Data collection	
Diffractometer	Bruker D8 Venture
Absorption correction	Multi-scan (*SADABS*; Bruker, 2021 ▸)
*T*_min_, *T*_max_	0.602, 0.650
No. of measured, independent and observed [*I* ≥ 2σ(*I*)] reflections	45327, 4856, 4785
*R*_int_	0.026
(sin θ/λ)_max_ (Å^−1^)	0.834

Refinement	
*R*[*F* ^2^ > 2σ(*F* ^2^)], *wR*(*F* ^2^), *S*	0.011, 0.024, 1.14
No. of reflections	4856
No. of parameters	221
H-atom treatment	H atoms treated by a mixture of independent and constrained refinement
Δρ_max_, Δρ_min_ (e Å^−3^)	0.14, −0.22
Absolute structure	Hooft *et al.*, 2010 ▸
Absolute structure parameter	−0.011 (7)

Computer programs: *APEX4* and *SAINT* (Bruker, 2021 ▸), *SHELXS* (Sheldrick, 2008 ▸), *SHELXL* (Sheldrick, 2015 ▸), *OLEX2* (Dolomanov *et al.*, 2009 ▸), *CrystalExplorer21* (Spackman *et al.*, 2021 ▸; Turner *et al.*, 2017 ▸), *publCIF* (Westrip, 2010 ▸), *Mercury* (Macrae *et al.*, 2020 ▸), *NoSpherA2* (Kleemiss *et al.*, 2021 ▸), *GaussView 6.016* (Frisch *et al.*, 2016 ▸), *Gaussian 09* (Frisch *et al.*, 2016 ▸), *Molekel 4.3* (Flükiger *et al.*, 2000 ▸) and *PLATON* (Spek, 2020 ▸).

## supplementary crystallographic information

### Crystal data

**Table:**

C_9_H_14_N^+^·Cl^−^	*D*_x_ = 1.141 Mg m^−^^3^
*M**_r_* = 171.67	Mo *K*α radiation, λ = 0.71073 Å
Orthorhombic, *P*2_1_2_1_2_1_	Cell parameters from 1282 reflections
*a* = 6.7723 (6) Å	θ = 3.2–30.5°
*b* = 7.1806 (5) Å	µ = 0.32 mm^−^^1^
*c* = 20.542 (2) Å	*T* = 100 K
*V* = 998.96 (16) Å^3^	Block, colourless
*Z* = 4	0.48 × 0.39 × 0.37 mm
*F*(000) = 368.574	

### Data collection

**Table:**

Bruker D8 Venture diffractometer	4785 reflections with *I*≥ 2σ(*I*)
Detector resolution: 10.4167 pixels mm^-1^	*R*_int_ = 0.026
ω and φ scans	θ_max_ = 36.4°, θ_min_ = 3.0°
Absorption correction: multi-scan (SADABS; Bruker, 2021)	*h* = −11→11
*T*_min_ = 0.602, *T*_max_ = 0.650	*k* = −11→11
45327 measured reflections	*l* = −34→34
4856 independent reflections	

### Refinement

**Table:**

Refinement on *F*^2^	Primary atom site location: iterative
Least-squares matrix: full	H atoms treated by a mixture of independent and constrained refinement
*R*[*F*^2^ > 2σ(*F*^2^)] = 0.011	*w* = 1/[σ^2^(*F*_o_^2^) + (0.0056*P*)^2^ + 0.0168*P*] where *P* = (*F*_o_^2^ + 2*F*_c_^2^)/3
*wR*(*F*^2^) = 0.024	(Δ/σ)_max_ = −0.001
*S* = 1.14	Δρ_max_ = 0.14 e Å^−^^3^
4856 reflections	Δρ_min_ = −0.22 e Å^−^^3^
221 parameters	Absolute structure: Hooft *et al.*, 2010
0 restraints	Absolute structure parameter: −0.011 (7)
0 constraints	

### Special details

**Table:**

Refinement. Refinement using *NoSpherA2*, an implementation of NOn-SPHERical Atom-form-factors in *Olex2*. 2021 *NoSpherA2* implementation of HAR makes use of tailor-made aspherical atomic form factors calculated on-the-fly from a Hirshfeld-partitioned electron density (ED) - not from spherical-atom form factors. Hydrogen atom H1b was refined isotropically and freely without using calculated ED. Distances and Angles concerning H1b were evaluated by PLATON.The ED is calculated from a gaussian basis set single determinant SCF wavefunction - either Hartree-Fock or DFT using selected funtionals - for a fragment of the crystal. This fragment can be embedded in an electrostatic crystal field by employing cluster charges or modelled using implicit solvation models, depending on the software used. The following options were used: SOFTWARE: *Olex2* 1.5 alpha, B1387_0m.wfn as wfn-file, PARTITIONING: *NoSpherA2*, INT ACCURACY: Normal, METHOD: PBE, BASIS SET: def2-SVP, CHARGE: 0, MULTIPLICITY: 1, DATE: 2021.12.08_16:09:30.

### Fractional atomic coordinates and isotropic or equivalent isotropic displacement parameters (Å^2^)

**Table:**

	*x*	*y*	*z*	*U*_iso_*/*U*_eq_	
Cl1	0.592521 (11)	0.543691 (10)	0.179385 (3)	0.016339 (15)	
N1	0.41684 (4)	0.47620 (3)	0.316327 (11)	0.01307 (4)	
H1a	0.4079 (9)	0.3344 (8)	0.3230 (3)	0.0295 (14)	
H1b	0.4763 (8)	0.4952 (8)	0.2706 (3)	0.0192 (14)*	
C1	0.09588 (5)	0.45714 (5)	0.262760 (15)	0.01939 (5)	
H1c	0.1708 (9)	0.4643 (10)	0.2151 (3)	0.0406 (16)	
H1d	0.0800 (10)	0.3125 (8)	0.2759 (3)	0.0423 (17)	
H1e	−0.0497 (8)	0.5137 (10)	0.2588 (3)	0.0407 (17)	
C2	0.21347 (4)	0.56000 (4)	0.314781 (13)	0.01314 (4)	
H2	0.2339 (7)	0.7048 (6)	0.3009 (2)	0.0231 (12)	
C3	0.55360 (4)	0.55695 (5)	0.365038 (15)	0.01794 (5)	
H3a	0.5664 (10)	0.7052 (8)	0.3574 (3)	0.0433 (17)	
H3b	0.4987 (8)	0.5322 (10)	0.4134 (3)	0.0409 (16)	
H3c	0.6979 (9)	0.4918 (9)	0.3592 (3)	0.0420 (18)	
C4	0.11585 (4)	0.55281 (5)	0.380803 (13)	0.01459 (4)	
C5	0.10068 (6)	0.38632 (5)	0.415554 (17)	0.02390 (6)	
H5	0.1630 (10)	0.2592 (8)	0.3963 (3)	0.0438 (17)	
C6	0.00496 (6)	0.38292 (6)	0.47550 (2)	0.02996 (8)	
H6	−0.0039 (10)	0.2515 (11)	0.5014 (4)	0.062 (2)	
C7	−0.07980 (5)	0.54394 (7)	0.500661 (15)	0.02628 (6)	
H7	−0.1515 (9)	0.5389 (10)	0.5478 (3)	0.0464 (17)	
C8	−0.06743 (5)	0.70924 (5)	0.465812 (16)	0.02150 (6)	
H8	−0.1335 (10)	0.8354 (8)	0.4853 (3)	0.0462 (18)	
C9	0.03231 (5)	0.71413 (5)	0.406506 (15)	0.01653 (5)	
H9	0.0436 (8)	0.8430 (8)	0.3791 (3)	0.0330 (15)	

### Atomic displacement parameters (Å^2^)

**Table:**

	*U*^11^	*U*^22^	*U*^33^	*U*^12^	*U*^13^	*U*^23^
Cl1	0.02160 (3)	0.01263 (2)	0.01478 (2)	−0.00082 (3)	0.00376 (2)	−0.00023 (2)
N1	0.01278 (8)	0.01374 (9)	0.01268 (8)	−0.00043 (8)	0.00098 (8)	−0.00014 (8)
H1a	0.028 (3)	0.022 (3)	0.038 (4)	−0.001 (3)	−0.002 (3)	0.005 (3)
C1	0.01783 (11)	0.02068 (12)	0.01965 (11)	−0.00110 (14)	−0.00559 (11)	−0.00209 (11)
H1c	0.034 (3)	0.067 (5)	0.021 (3)	−0.008 (4)	−0.006 (3)	−0.008 (3)
H1d	0.051 (4)	0.033 (4)	0.043 (4)	−0.011 (3)	−0.024 (4)	0.002 (3)
H1e	0.020 (3)	0.055 (5)	0.047 (4)	0.007 (3)	−0.014 (3)	−0.004 (3)
C2	0.01310 (9)	0.01303 (10)	0.01329 (10)	−0.00042 (8)	−0.00041 (8)	0.00049 (9)
H2	0.023 (3)	0.016 (3)	0.031 (3)	−0.003 (2)	−0.003 (2)	0.003 (2)
C3	0.01450 (11)	0.01998 (13)	0.01934 (12)	−0.00051 (10)	−0.00309 (9)	−0.00190 (11)
H3a	0.049 (4)	0.016 (3)	0.065 (4)	−0.005 (3)	−0.015 (4)	−0.003 (3)
H3b	0.030 (3)	0.065 (5)	0.028 (3)	−0.009 (4)	−0.001 (3)	0.001 (3)
H3c	0.026 (3)	0.059 (5)	0.041 (4)	0.015 (3)	−0.003 (3)	−0.015 (3)
C4	0.01291 (10)	0.01579 (11)	0.01505 (10)	0.00125 (10)	0.00171 (8)	0.00135 (10)
C5	0.02478 (14)	0.02138 (14)	0.02555 (14)	0.00664 (13)	0.01142 (13)	0.00891 (12)
H5	0.059 (4)	0.028 (3)	0.045 (4)	0.009 (3)	0.020 (3)	0.014 (3)
C6	0.02922 (17)	0.03372 (19)	0.02696 (17)	0.00858 (15)	0.01400 (14)	0.01358 (15)
H6	0.071 (5)	0.050 (4)	0.066 (4)	0.026 (4)	0.034 (4)	0.036 (4)
C7	0.02098 (13)	0.03984 (18)	0.01803 (12)	0.00485 (17)	0.00607 (11)	0.00307 (14)
H7	0.049 (4)	0.062 (4)	0.028 (3)	0.005 (4)	0.020 (3)	0.005 (3)
C8	0.01775 (13)	0.02930 (15)	0.01743 (12)	0.00201 (12)	0.00142 (10)	−0.00627 (12)
H8	0.058 (5)	0.036 (4)	0.045 (4)	0.009 (3)	0.016 (4)	−0.011 (3)
C9	0.01530 (11)	0.01815 (13)	0.01614 (11)	0.00065 (10)	−0.00044 (9)	−0.00345 (10)
H9	0.026 (3)	0.034 (3)	0.039 (3)	0.001 (3)	0.006 (3)	0.003 (3)

### Geometric parameters (Å, º)

**Table:**

N1—H1a	1.029 (6)	C3—H3c	1.090 (6)
N1—H1b	1.031 (6)	C4—C5	1.3962 (4)
N1—C2	1.5034 (4)	C4—C9	1.3931 (4)
N1—C3	1.4816 (4)	C5—H5	1.080 (6)
C1—H1c	1.104 (6)	C5—C6	1.3919 (5)
C1—H1d	1.079 (6)	C6—H6	1.085 (7)
C1—H1e	1.070 (5)	C6—C7	1.3905 (6)
C1—C2	1.5237 (4)	C7—H7	1.084 (5)
C2—H2	1.087 (4)	C7—C8	1.3886 (6)
C2—C4	1.5097 (4)	C8—H8	1.087 (6)
C3—H3a	1.080 (6)	C8—C9	1.3935 (5)
C3—H3b	1.075 (5)	C9—H9	1.086 (6)
			
H1b—N1—H1a	106.0 (5)	H3c—C3—N1	108.5 (3)
C2—N1—H1a	110.2 (3)	H3c—C3—H3a	109.6 (5)
C2—N1—H1b	106.6 (3)	H3c—C3—H3b	109.9 (4)
C3—N1—H1a	109.5 (3)	C5—C4—C2	121.39 (3)
C3—N1—H1b	108.6 (3)	C9—C4—C2	119.33 (3)
C3—N1—C2	115.49 (2)	C9—C4—C5	119.23 (3)
H1d—C1—H1c	108.3 (5)	H5—C5—C4	120.5 (3)
H1e—C1—H1c	109.8 (4)	C6—C5—C4	120.11 (3)
H1e—C1—H1d	107.0 (5)	C6—C5—H5	119.4 (3)
C2—C1—H1c	111.1 (3)	H6—C6—C5	118.4 (4)
C2—C1—H1d	110.0 (3)	C7—C6—C5	120.44 (4)
C2—C1—H1e	110.6 (3)	C7—C6—H6	121.2 (4)
C1—C2—N1	107.44 (2)	H7—C7—C6	119.3 (4)
H2—C2—N1	105.8 (3)	C8—C7—C6	119.62 (3)
H2—C2—C1	110.3 (3)	C8—C7—H7	121.1 (4)
C4—C2—N1	111.62 (2)	H8—C8—C7	119.9 (3)
C4—C2—C1	112.62 (2)	C9—C8—C7	120.10 (3)
C4—C2—H2	108.9 (2)	C9—C8—H8	120.0 (3)
H3a—C3—N1	109.7 (3)	C8—C9—C4	120.48 (3)
H3b—C3—N1	110.1 (3)	H9—C9—C4	118.9 (3)
H3b—C3—H3a	109.0 (5)	H9—C9—C8	120.6 (3)

### Hydrogen-bond geometry (Å, º)

**Table:**

*D*—H···*A*	*D*—H	H···*A*	*D*···*A*	*D*—H···*A*
N1—H1*a*···Cl1^i^	1.029 (6)	2.088 (6)	3.1075 (3)	170.4 (5)
N1—H1*b*···Cl1	1.031 (6)	2.062 (6)	3.0925 (4)	177.8 (5)

Symmetry code: (i) −*x*+1, *y*−1/2, −*z*+1/2.
